# Formula feeding and associated factors among mothers with infants 0–6 months old in Mettu Town, Southwest Ethiopia

**DOI:** 10.1002/fsn3.3403

**Published:** 2023-05-02

**Authors:** Abeza Mitiku Kera, Asrat Zewdie, Wakuma Akafu, Radiet Kidane, Meseret Tamirat

**Affiliations:** ^1^ Department of Public Health, College of Health Science Mattu University Mettu Ethiopia; ^2^ Department of Health Service Management, Faculty of Public Health, Institute of Health Jimma University Jimma Ethiopia; ^3^ Department of Nutrition and Dietetics, Faculty of Public Health, Institute of Health Jimma University Jimma Ethiopia

**Keywords:** Ethiopia, formula feeding, infants aged 0–6 months, Mettu Town

## Abstract

Adequate nutrition during infancy is essential for children's normal development and well‐being. However, the duration of breastfeeding has been declining and is being replaced by formula feeding, particularly in the urban communities of developing countries, including Ethiopia. Hence, this study aimed to assess formula feeding and its associated factors, as relatively little information is available regarding this problem in Ethiopia, particularly in Mettu Town. A community‐based cross‐sectional study was conducted in Mettu Town from May 17 to July 1, 2021, among 366 mothers with infants 0–6 months old. A simple random sampling technique was used in this study. Pre‐tested semi‐structured questionnaires were used to collect the data. Descriptive statistics and multivariable logistic regression were performed, and variables with a p‐value <0.05 in the final model were declared statistically significant with formula feeding found to be 28.4% [95% CI: (24.0–33.0)]. Primiparity [AOR = 3.27, 95% CI: (1.71–6.27)], cesarean delivery [AOR = 2.62, 95% CI: (1.28–5.35)], initiation of breastfeeding after 24 h [AOR = 3.5, 95% CI: (1.74–10.0)], employed mothers [AOR = 2.4, 95% CI: (1.29–4.19)], positive attitude toward formula feeding [AOR = 2.4, 95% CI: (1.29–4.19)], and poor knowledge of formula feeding [AOR = 2.6, 95% CI (1.49–4.74)] were factors significantly associated with formula feeding. Almost one‐third of the mothers were formula feeding their infants. Primiparity, maternal employment, initiation of breast milk after 24 h, cesarean delivery, poor maternal knowledge, and positive attitude toward formula feeding were among the contributing factors to this high formula‐feeding practice. Hence, much effort should be invested in educating pregnant and lactating mothers to improve their knowledge of formula feeding while working on activities that change their attitude toward formula feeding.

## Graphical abstract

According to the study, nearly half of the mothers were practicing formula feeding for their children.Being a primipara, being an employed mother, having poor knowledge of formula milk, and positive attitude toward formula were factors associated with formula feeding.

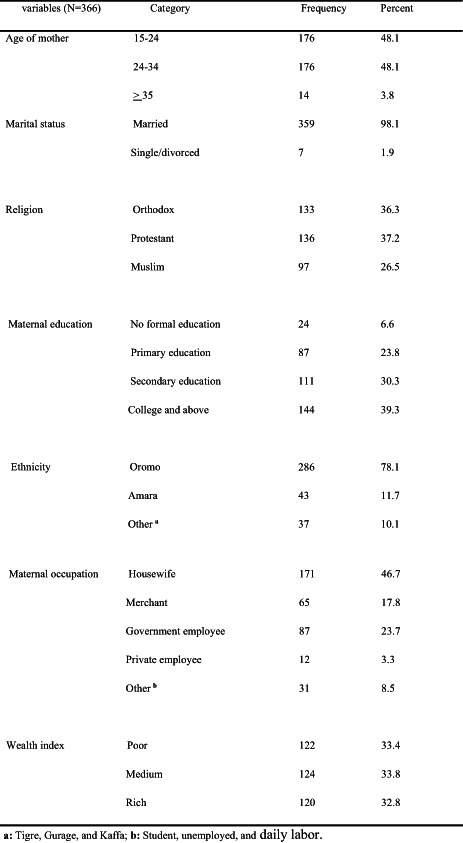

## 1 INTRODUCTION

Infancy is a critical period in which rapid physical growth, as well as cognitive and emotional development, occurs. Adequate nutrition during infancy is essential for normal development and well‐being (UNICEF, 2019). Breast milk is universally recommended as the best and the most complete food for infants. Thus, early initiation of breastfeeding within 1 h of birth, exclusive breastfeeding of infants for the first 6 months of life, and continued breastfeeding with appropriate complementary foods for 2 or more years are the basis for growth and development (Baker et al., 2020; WHO Secretariat, 2010). Although breastfeeding is strongly advocated, there is a shift in exclusive breastfeeding practices toward the introduction of formula feeding, particularly in industrialized countries and urban communities of developing countries, including Ethiopia (Leshi & Sanusi, 2014; Tawfik et al., 2019; Taye et al., 2021).

Owing to the effects of globalization, urbanization, changes in the nature of women's work, increased availability of formula milk in supermarkets, and promotion of breast milk substitutes through advertising in various media, the proportion and duration of breastfeeding are declining and being replaced by formula feeding (Baker et al., 2020). Infants on formula feeding are more vulnerable to childhood infectious diseases such as otitis media, gastroenteritis, and pneumonia. Formal‐fed infants are also at a higher risk of developing non‐communicable diseases such as diabetes mellitus, decreased cognitive development, and an increased risk of obesity later in life (Baker et al., 2020; Victora et al., 2016).

Since 1981, the World Health Organization (WHO) has enacted several legal measures regarding the international marketing of breast milk substitutes (BMSs). However, according to the WHO, 2020 report, only 136 (70%) of 194 WHO member states (countries) have enacted legal measures with provisions to implement the code (WHO, 2020).

Since 2016, the Ethiopian government has implemented various guidelines to support breastfeeding and guide its promotion of BMS, including the “Infant Formula and Follow‐up Formula Directive No. 30/2016” and the “Food Advertisement Directive 33/2016” (Laillou et al., 2021). However, according to studies conducted in Dire‐Dawa (Dagne et al., 2019), Jimma, and Addis Ababa, 21.4%, 61.9%, and 46.2% of infants aged within 6 months had been fed infant formula, respectively (Abebe et al., 2019; Ratna & Wahyu, 2018; Taye et al., 2021).

Maternal occupation, educational status, maternal health status, delivery by cesarean section (C/S), attitude toward formula, milk advertisement, and economic factors have an impact on mothers feeding their infants with infant formula (Legesse et al., 2014; Pries et al., 2016; Taye et al., 2021). Parents may not know about the nutritional content of formula milk and the health impact of feeding their infants with formula milk. Few studies have been conducted regarding formula feeding in Ethiopia, and no similar study has been conducted in Mettu Town. Hence, this study aimed to assess the status and factors influencing formula feeding among mothers with infants from 0 to 6 months old in Mettu Town, which could help program designers, healthcare managers, and healthcare workers to know the level of formula feeding and contributing factors in the study area to design and implement behavioral change interventions on modifiable contributing factors to formula feeding.

## 2 MATERIALS AND METHODS

### 2.1 Study setting and period

The study was conducted in Mettu Town, and data were collected from May 17 to July 1, 2021. Mettu Town has located 600 km from Addis Ababa, the capital city of the country. The town is divided into three kebeles, with an estimated total population of 49,538 and 10,321 households, respectively. The estimated number of women in the reproductive‐age group was 9214. The town has one public hospital, one health center, and three health posts with 12 health extension workers, 11 medium private clinics, 6 private small clinics, 12 drug vendors, and 3 pharmacies.

### 2.2 Study design and population

A community‐based cross‐sectional study design was used. All mothers with index infants aged 0–6 months and living in Mettu Town were the source population, whereas mothers with infants aged 0–6 months and selected by simple random sampling techniques were the study population.

### 2.3 Eligibility criteria

All mothers with 0‐ to 6‐month‐old infants who had lived for at least 6 months in the town were included in the study. Mothers who used infant formula for medical reasons and those who were unable to complete the interview because of severe medical or psychiatric problems were excluded from the study.

### 2.4 Sample size determination and sampling procedure

The required sample size was determined using a single population proportion formula by considering the proportion of formula feed as 61.9% (Ratna & Wahyu, 2018), a level of confidence of 95%, and a margin of error of 5%. The initial sample size was 362. Considering a 5% non‐response rate, the final sample size was found to be 380.

Before data collection, a list of mothers who gave live births within a 6‐month interval was collected from the delivery books of both public and private health institutions in the town. Accordingly, we found 659 mothers who gave birth within a 6‐month interval in Mettu Town. A sampling frame was prepared, and the required number of mothers to be selected from each kebele was determined based on proportion to population size allocation. Finally, study participants were selected using a computer‐generated simple random sampling technique.

### 2.5 Data collection tools and techniques

Data were collected through face‐to‐face interviews using a pre‐tested semi‐structured questionnaire. The questionnaires were adapted from related articles (Pries et al., 2016; Ratna & Wahyu, 2018; Tawfik et al., 2019; Taye et al., 2021). The questionnaire had five parts: socioeconomic and demographic characteristics, maternal health service and child characteristics, maternal knowledge of formula feeding, maternal attitude toward formula feeding, and formula feeding practices of mothers.

The questionnaires were first prepared in English and then translated into Afan Oromo (local language) and translated back to English by an expert who had a good ability in the two languages to check consistency. Before data collection, the questionnaires were pre‐tested on a 5% sample size in a nearby town, which was not a part of the actual data collection. Based on the pre‐test, some modifications, such as unclear or vague questions and incorrect skip patterns, were corrected. The internal consistency of the independent variables with the 5‐point Likert scale was checked using Cronbach's alpha, which was found to be 0.72.

Data were collected by six urban Health Extension Workers using pre‐tested and semi‐structured questionnaires through house‐to‐house visits by mothers with an index of infants aged 0–6 months. Two professional nurses were recruited as supervisors during the data collection. If an eligible mother was absent from the home at the time of data collection, a revisit was performed three times, and the mother who was absent at the third visit was considered a non‐respondent.

Two days of training were provided to data collectors and supervisors by the principal investigator on data collection tools, data collection techniques, approach to the interviews, and maintaining the privacy and confidentiality of the respondents. Every day after data collection, the questionnaires were reviewed and checked for completeness by supervisors and the principal investigator.

### 2.6 Operational definition

Formula feeding: Feeding formula milk to an infant under the aged of 0–6 months via a bottle, rubber, nipples, or cups as a supplement to breast milk or as a complete breast milk substitute during the last 24 h. It was measured by a “Yes” (1) and “No” (2) question. The mothers who answered “Yes” to the question were considered mothers who fed their infants with formula milk (Yes was coded as 1 and No was given a code of 0 for multivariable logistic regression) (Abebe et al., 2019).

#### 2.6.1 Antenatal service utilization

At least one visit to a health facility for checkup purposes during pregnancy of the current index child (Legesse et al., 2014).

#### 2.6.2 Attitude toward formula feeding

Respondents were asked a set of questions containing seven items that ranged from strongly disagree to strongly agree using a 5‐point Likert scale. When the total score of each respondent is close to 35, it indicates the most favorable attitude, and when the score is close to 7, it indicates the most unfavorable attitude toward formula feeding. Based on the total summation of the scales, respondents were classified as having a negative attitude toward formula feeding if they scored less than 21, neutral if they scored 21, or a positive attitude score above 21 (Akafu & Geta, 2020).

#### 2.6.3 Knowledge of formula feeding

This was measured using a yes/no dichotomous questionnaire that consisted of six items. The answers to each question were analyzed as known and unknown. Each correct answer was coded as “1”, which is known, and each wrong answer was “0” (do not know). The maximum attainable score was 6, and the minimum possible score was 0. Respondents whose knowledge scores were equal to or above the mean were categorized as having good knowledge, whereas those scoring below the mean score were categorized as having poor knowledge of formula feeding (Mekuria & Edris, 2015).

#### 2.6.4 Wealth index

Household assets were assessed using 22 items. Principal component analysis (PCA) was used to derive factor scores, and the composite score was then ranked into three tertiles. The first tertile was classified as poor and the last as rich (Ethiopia Demographic and Health Survey, 2016).

### 2.7 Data quality, processing, and analysis

For effective and quality data collection, experts assessed whether the data collection tool measured what it was intended to measure and if it was comprehensive enough to collect all the information needed to address the objective of the study. The reliability of the questionnaire was assessed. Training was provided to data collectors and supervisors. A pre‐tested tool was used for data collection, and the non‐response rate was reduced by repeated contact. All data were visually checked, coded, and entered into the Epi‐data version 4.6 before being exported to the SPSS version 26 software package for analysis. After categorizing and defining the variables, descriptive analysis was performed for each of the independent variables using frequencies, cross‐tabulation, and percentages.

Before performing PCA for variables explaining the household wealth index, all necessary assumptions and prerequisites were checked appropriately. The degree of association between independent and dependent variables was assessed using the adjusted odds ratio (AOR) with a 95% confidence interval. Simple binary logistic regression analysis was performed to select candidate variables for multivariable analysis. A variable with a *p*‐value <.25 was taken as a candidate for the multiple regression analysis, and a *p*‐value <.05 was declared as statistically significant in the final model. Pseudo‐regression was performed to check for multicollinearity between the independent variables. The minimum tolerance and maximum variance inflation (VIF) factors were found to be 0.60 and 1.69, respectively. For the final fitted multivariable logistic regression model, the adequacy of the model to predict the outcome variables was checked using the Hosmer–Lemeshow goodness of fit, and the *p‐*value was found to be >.05.

## 3 RESULTS

### 3.1 Socio‐economic and demographic characteristics of participants

A total of 366 mothers with infants aged 0–6 months participated in the study, with a response rate of 96.3%. The mean age of the respondents was 25.5 years (SD ± 4.4), ranging from a minimum age of 18 years to a maximum age of 39. Almost all (98.1%) of the mothers were married. By religion, 37.2% were Protestant and 78.1% were Oromo. Regarding educational status and occupation, 6.6% had no formal education, 39.3% had a college degree or above, and 46.7% were housewives. In terms of wealth status, one‐third of the respondents (33.4%) ranked poor (Table 1).

**TABLE 1:** Socio‐demographic characteristics of mothers with infants aged 0–6 months old, Mettu Town, Southwest Ethiopia, 2021.

Variables (*N* = 366)	Category	Frequency	Percent
Age of mother	15–24	176	48.1
	24–34	176	48.1
	≥35	14	3.8
Marital status	Married	359	98.1
	Single/divorced	7	1.9
Religion	Orthodox	133	36.3
	Protestant	136	37.2
	Muslim	97	26.5
Maternal education	No formal education	24	6.6
	Primary education	87	23.8
	Secondary education	111	30.3
	College and above	144	39.3
Ethnicity	Oromo	286	78.1
	Amara	43	11.7
	Other^a^	37	10.1
Maternal occupation	Housewife	171	46.7
	Merchant	65	17.8
	Government employee	87	23.7
	Private employee	12	3.3
	Other^b^	31	8.5
Wealth index	Poor	122	33.4
	Medium	124	33.8
	Rich	120	32.8

^a^ Tigre, Gurage, and Kaffa.

^b^ Student, unemployed, and daily labor.

### 3.2 Maternal health service utilization and infant‐related characteristics

Regarding the obstetric experience of the participants, 57.4% of them were multiparas. Almost all (99.5%) participants get at least one ANC follow‐up. However, only 15.8% of patients had four or more ANC visits. Regarding infant feeding counseling during ANC, 60.4% of them were counseled on infant feeding. The majority of the study participants (92.6%) gave birth at public health institutions, and 80.3% were delivered spontaneously or vaginally. The majority (88.3%) received postnatal care within 24 h of delivery.

All infants were breastfed at any time, and 76.5% of the mothers initiated breastfeeding within 1 h of delivery. The mean age of the infants was 3.2 months (SD ± 1.4), with a minimum of 1 month and a maximum of 6 months (Table 2).

**TABLE 2:** Maternal health service utilization and infant characteristics among mothers with infants 0–6 months old, Mettu Town, Southwest Ethiopia, 2021.

Variable (*N* = 366)	Category	Frequency	Percent
Parity	Primipara	156	42.6
	Multipara	210	57.4
ANC follow up	Yes	364	99.5
	No	2	0.5
Number of visits	<4 visit	308	84.2
	≥4 visit	58	15.8
Counseling on infant feeding	Yes	221	60.4
	No	145	39.6
Place of delivery	Public health institution	339	92.6
	Private health institution	27	7.4
Mode of delivery	Normal/Vaginal	294	80.3
	C/S	72	19.7
PNC care	Yes	323	88.3
	No	43	11.7
Age of the infant	<2 months	113	30.9
	2–3 months	134	36.6
	4–6 months	119	32.5
Sex of infant	Male	178	48.6
	Female	188	51.4
Ever breastfed	Yes	366	100
	No	0	0
Timing of breast milk initiation	Within 1 h	280	76.5
	1‐23 h	59	16.1
	After 24 h	27	7.4

Abbreviations: ANC, antenatal care; C/S, cesarean section; PNC, postnatal care.

### 3.3 Maternal knowledge of formula feeding

In terms of respondents' knowledge of infant formula, 81.4% had heard about it, and the most common source of information was from their peers or neighbors (70.1%), followed by TV/radio (42.6%). Among them, 43.3% of the respondents said infant formula increased a baby's immunity, while 60.1% said infant formula contributed to a baby's brain development. Almost 14% of the respondents said infant formula is more nutritious than breast milk, and 68.8% of the respondents do not know the future health impact of formula feeding. The mean score was 2.9 with an SD of ±1.35. Based on the mean knowledge score, 46.1% of the participants were categorized as having poor knowledge and 53.9% have good knowledge of formula feeding (Table 3).

**TABLE 3:** Maternal knowledge of formula feeding among mothers with infants 0–6 months old, Mettu Town, Southwest Ethiopia, 2021.

Question	Response	Frequency	Percent
Have you ever heard about infant formula	Yes	298	81.4
	No	69	18.9
Source information	Peer/neighbors	209	70.1
	TV/Radio	127	42.6
	Family	55	18.5
	Health professional	50	16.8
Does infant formula increase your baby's immunity?	Yes	129	43.3
	No	169	56.7
Does formula feeding contribute to child brain development	Yes	181	60.7
	No	117	39.3
Does formula feeding increase the risk of diarrhea and other infection	Yes	107	35.9
	No	191	64.1
Does formula feeding increase the risk of childhood obesity	Yes	131	44
	No	167	56
Is infant formula more nutritious than breast milk	Yes	41	13.8
	No	257	86.2
Does formula feeding affect the future health status of the baby	Yes	93	31.2
	No	205	68.8

### 3.4 Maternal attitude toward formula feeding

Regarding the attitudes of respondents toward infant formula, 35.9% disagreed that formula feeding ensures optimal health for the baby, while 48% agreed. Among the study participants, 79.9% disagreed with the idea that formula feeding is more convenient than breast milk feeding, while 6.4% agreed. One‐third of the respondents disagreed with the idea that infant formula should be advised when a baby's growth is sluggish or weak, while 44% agreed. Two‐thirds of the participants disagreed with the idea that the baby was willing to feed formula milk rather than breast milk, while 8.7% agreed. Sixty‐two percent of participants disagree with the idea that formula feeding gives you more comfort than breastfeeding, while 13.1% agreed. Based on the summation of the scales, 63.1% of the participants were categorized as having a negative attitude, whereas 12.4% and 24.5% were categorized as having neutral and positive attitudes, respectively (Table 4).

**TABLE 4:** Maternal attitude toward formula feeding among mothers with infants 0–6 months old, Mettu Town, Southwest Ethiopia, 2021.

Variables	Responses		
	Disagree	Neutral	Agree
Formula feeding ensures optimal health for the baby	107 (35.9%)	48 (16.1%)	143 (48%)
It is more convenient than breast milk	238 (79.9%)	41 (13.8%)	19 (6.4)
It is advised when the baby's growth is sluggish/weak	95 (31.9%)	72 (24.2)	131 (44%)
Your baby is willing to feed formula milk than breast milk	198 (66.4%)	74 (24.8%)	26 (8.7%)
It gives you more comfort than breastfeeding	186 (62.4%)	73 (24.5%)	39 (13.1%)
It makes your baby smart	107 (35.9%)	144 (48.3%)	47 (15.8%)
Rich family feed their baby with formula milk while poor provides only breast milk	132 (44.3%)	110 (36.9%)	56 (18.8)

### 3.5 Formula feeding and related characteristics

Among the total study participants, 28.4% [95% CI: 24.0–33.0] formula fed their babies. Among the respondents practicing formula feeding, 95.2% of them were feeding combinations of both breast milk and formula, whereas only 4.8% were feeding their babies with only formula milk. The main reasons for the initiation of infant formula were breast milk insufficiency (42.3%), being busy with work (34.6%), and sluggish growth of the baby (23.1%). The average age of initiating infant formula was 2.3 months, SD of ±1.04, which was found within 2–3 age intervals.

### 3.6 Factors associated with formula feeding

On multivariable logistic regression analysis, primipara mothers (AOR = 3.27, 95% CI:1.71–6.27), delivery by cesarean section (AOR = 2.62, 95% CI:1.28–5.35), and initiation of breast milk after 24 h (AOR = 3.5, 95% CI:1.74–10.0), employed mothers (AOR = 2.4, 95% CI: 1.29–4.19), poor knowledge of formula feeding (AOR = 2.6, 95% CI:1.27–4.74), and positive attitude toward formula feeding (AOR = 2.4, 95% CI:1.49–4.74) were significantly associated with formula feeding (Table 5).

**TABLE 5:** Multivariable logistic regression analysis of factors associated with formula feeding among mothers with infants 0–6 months old in Mettu Town, Southwest Ethiopia, 2021.

Variable	Formula feeding			
	Yes (%)	No (%)	COR (95% CI)	AOR (95% CI)
Age of mother				
15–24	34 (19.3)	142 (80.7)	0.31 (0.104–0.98)	0.4 (0.09–1.71)
25–34	64 (36.4)	112 (63.6)	0.762 (0.25–2.29)	1.2 (0.28–4.82)
35 above	6 (42.9)	8 (57.1)	1	
Maternal education				
No formal education	2 (8.3)	22 (91.7)	1	
Primary education	22 ((25.3)	65 (74.7)	3.7 (0.80–17.12)	3.5 (0.65–19.81)
Secondary	30 (27)	81 (73)	4.2 (0.90–18.38)	4.5 (0.83–24.81)
College/above	50 (34.7)	94 (65.3)	5.8 (1.32–25.90)	2.3 (0.39–13.58)
Employment status				
Unemployed	60 (22.5)	207 (77.5)	1	
Employed	44 (44.4)	55 (55.6)	2.7 (1.69–4.50)	2.4 (1.29–4.19)**
Wealth status				
Poor	22 (18.9)	99 (81.1)	1	
Medium	38 (30.6)	86 (69.4)	1.9 (1.05–3.44)	1.18 (0.55–2.52)
Rich	43 (35.80	77 (64.2)	2.4 (1.33–4.32)	2 (0.93–4.43)
Parity				
Primipara	55 (35.3)	101 (64.7)	1.8 (1.02–2.83)	3.27 (1.71–6.27)**
Multipara	49 (23.3)	161 (76.7)	1	
Frequency of ANC visit				
<4 visits	94 (30.5)	214 (69.5)	2.1 (1.02–4.34)	2 (0.84–5.33)
≥4 visits	10 (17.2)	48 (82.8)	1	
Mode of delivery				
Spontaneously	62 (21.1)	232 (78.9)	1	
C/s	42 (58.3)	30 (41.7)	5.2 (3.03–9.04)	2.62 (1.28–5.35)**
Timing of breast milk initiation				
Within 1 h	64 (22.9)	216 (77.1)	1	
1–23 h	24 (40.70	35 (59.3)	2.3 (1.28–4.17)	1.5 (0.74–3.18)
After 24 h	16 (59.3)	11 (40.7)	4.9 (2.16–11.11)	3.5 (1.74–10.0)**
Sex of infant				
Male	61 (34.3)	117 (65.7)	1.6 (1.05–2.82)	1.5 (0.87–2.82)
Female	43 (22.9)	145 (77.1)	1	
Age of infant				
>2 months	22 (19.5)	91 (80.5)	1	
2–3 months	42 (31.3)	92 (68.7)	1.8 (1.04–3.41)	1.3 (0.6–2.72)
4–6 months	40 (33.6)	79 (66.4)	2.2 (1.14–3.82)	2 (0.9–4.56)
Knowledge				
Poor	60 (44.4)	75 (55.6)	2 (1.27–3.37)	2.6 (1.27–4.74)**
Good	44 (27.8)	114 (72.2)	1	
Attitude				
Negative	58 (30.9)	130 (69.1)	1	
Neutral	13 (35.1)	24 (64.9)	1.4 (0.57–2.57)	1.7 (0.74–4.07)
Positive	33 (45.2)	40 (54.8)	1.8 (1.06–3.22)	2.4 (1.49–4.74)**

Abbreviations: AOR, adjusted odds ratio; COR, crude odds ratio.

** Statistically significant variables in multivariable logistic regression at *p*‐value <.05.

## 4 DISCUSSION

This study attempted to assess the prevalence of formula feeding and associated factors among mothers of infants aged 0–6 months in Mettu Town. Accordingly, this study found that 28.4% of mothers in the study area were formula feeding their babies. The prevalence of formula feeding in this study is comparable with a study conducted in Agaro Town, which was found to be 29% (Seid et al., 2019), and Dire‐Dawa, which was 21.4% (Dagne et al., 2019), whereas it was higher compared to the study conducted at Holeta (13.5%) (Kebebe & Assaye, 2017) and Gonder (12.4%) (Asfaw Admasu, 2016). The prevalence of formula feeding in this study was lower compared to the study conducted in Jimma (65%) (Abebe et al., 2019), Adis Abeba 47% (Taye et al., 2021), China 88% (Tang et al., 2015), Staten Island, New York 65% (Pierro et al., 2016), Pakistan 38% (Ijaz et al., 2015), Poland 42% (Rozensztrauch et al., 2021), East Malaysia 73.7% (Yee & Chin, 2007), Egypt 47% (Tawfik et al., 2019), and Nigeria 38.3% (Leshi & Sanusi, 2014). The inconsistency could be attributed to the result of differences in the current study's operational definition of formula feeding, socioeconomic status differences among study participants, and study period. A difference in marketing legislation of breast milk substitutes between the countries could also be a reason for this disparity.

The main reasons for the initiation of formula feeding in this study were insufficient breast milk (42.3%), being busy with work (34.6%), and sluggish baby growth (23.1%). Similar to studies conducted in Dire‐Dawa that reported breast milk insufficiency (85%) and busy with work (10%) (Dagne et al., 2019), Hangzhou and Shenzhen, China, reported perceived insufficient breast milk (86.2%), followed by a return to work (24.6%) (Tang et al., 2014), while in Poland, 62% of participants reported perceived insufficient breast milk (Rozensztrauch et al., 2021).

A study conducted in Egypt reported various maternal reasons, including insufficient breast milk at 60.9%, babies unable to suck at 50%, and 37% encouraged by healthcare providers (Tawfik et al., 2019). This indicates that there were different perceived reasons for the initiation of formula milk consumption in different places. This disparity could be attributed to differences in culture, methods of formula milk advertising, and sociodemographic characteristics of the study participants.

Regarding predictors of formula feeding, primipara mothers were more likely to practice formula feeding than multipara mothers. This finding is consistent with those of studies conducted in Taiwan (Chang et al., 2019), Malaysia (Tan, 2009), and Egypt (El Etreby et al., 2018). This might be due to a lack of knowledge regarding the benefits of breastfeeding among primipara women. In addition, postpartum primiparous mothers may face problems such as difficulty with motherhood and worry about their body image. The presence of this stress may lead to insufficient breast milk secretion (Elwelely & Mansour, 2018).

In the present study, the mode of delivery was significantly associated with formula feeding. Mothers who gave birth via caesarian section were more likely to initiate formula feeding for their babies than mothers who gave birth vaginally. The finding of this study is in line with those studies conducted in Dire‐Dawa (Dagne et al., 2019), Addis Abeba (Taye et al., 2021), Egypt (El Etreby et al., 2018), Western Nepal (Khanal et al., 2016), Indonesia (Nasrul et al., 2020), and North Jordan (Khasawneh & Khasawneh, 2017). This could be due to maternal stress‐related fear of the surgical procedure, which may reduce breast milk secretion by impairing oxytocin secretion (Dimitraki et al., 2016).

Mothers who initiated breastfeeding after 24 h of delivery were more likely to initiate formula feeding compared to those who initiated breastfeeding within 1 h of delivery. This finding is consistent with that of a study conducted in Addis Ababa (Taye et al., 2021). A possible reason might be that, as the time interval between delivery and initiation of breastfeeding increases, there is a probability for the initiation of pre‐lacteal feeding, which in turn leads to decreased lactogenesis and inadequate breast milk secretion.

Maternal employment status was significantly associated with formula feeding. Employed mothers were more likely to initiate formula than unemployed mothers. This finding is consistent with studies conducted in Indonesia (Nasrul et al., 2020), India (Rynjah et al., 2021), Namibia (Berde, 2017), North Jordan (Khasawneh & Khasawneh, 2017), and Gonder Town (Asfaw Admasu, 2016). This could be due to employed mothers being scheduled more on their outside work and having no adequate time for their baby to breastfeed. In addition, the absence of private breastfeeding or pumping areas in their workplace could be a reason for formula feeding. Contrary to this finding, a study conducted in Sokoharjo, Central Java, reported that working mothers had two times less chance of formula feeding their babies (Nuralita et al., 2017). The reason for this discrepancy might be due to the presence of a well‐established policy that supports breastfeeding at their workplace and the maternal experience of pumping breast milk and storing it for their babies before going to their workplace.

In this study, maternal knowledge of formula milk was significantly associated with formula feeding. Mothers who had poor knowledge of formula milk were 2.6 times more likely to practice formula feeding than those with good knowledge. This finding is similar to that of the study in Jimma (Abebe et al., 2019). This might be because mothers with poor knowledge of formula feeding would have a probability of inadequate breastfeeding knowledge that leads them to initiate formula milk for their babies. Mothers who had inadequate knowledge of infant formula were more likely to be shaped by the effects of infant formula promotion that derives them to formula feeding practices (Ratna & Wahyu, 2018).

Maternal attitude toward formula feeding was one of the predictors of formula feeding. Mothers who had a positive attitude toward formula feeding were two times more likely to give formula milk to their babies compared to those who had a negative or neutral attitude toward formula feeding, which is consistent with studies conducted in Jimma (Abebe et al., 2019) and Sukoharjo, Central Java (Nuralita et al., 2017). This might be because a woman who has a positive attitude toward formula feeding is easily derived to initiate formula milk to their babies when they find well‐packaged formula milk at pharmacies and supermarkets (Yee & Chin, 2007).

### 4.1 Limitations of the study

This study had certain limitations. First, since the data were collected through the verbal reports of the participants, there might be a possibility of response bias even though data collectors were trained in some techniques to reduce response bias. Second, the study did not assess formula‐feeding practice through the qualitative data collection method, as the method of data collection through a structured questionnaire might not allow participants to give their real practice and aggravating factors regarding the subject of formula feeding.

## 5 CONCLUSION

The current study reported that almost one‐third of mothers were feeding their infants with formula milk. Cesarean delivery, primiparity, timing of breast milk initiation, maternal employment status, poor maternal knowledge about formula feeding, and positive attitude toward formula feeding were found to be significantly associated with formula‐feeding practices. Hence, education on infant feeding, specifically the benefits of breast milk and demerits of formula feeding for all pregnant and lactating mothers, and encouragement of early initiation of breastfeeding after delivery for primipara mothers as well as mothers who gave birth by cesarean section should be implemented at the health facilities and community level.

## AUTHOR CONTRIBUTIONS

**Abeza Mitiku Kera:** Conceptualization (equal); formal analysis (equal); methodology (equal); validation (equal); visualization (equal); writing – original draft (equal). **Asrat Zewdie:** Conceptualization (equal); resources (equal); supervision (equal); validation (equal); visualization (equal). **Wakuma Akafu:** Conceptualization (equal); data curation (equal); methodology (equal); supervision (equal); validation (equal); visualization (equal). **Radiet Kidane:** Data curation (equal); resources (equal); supervision (equal); visualization (equal); writing – review and editing (equal). **Meseret Tamirat:** Data curation (equal); methodology (equal); resources (equal); visualization (equal); writing – review and editing (equal).

## FUNDING INFORMATION

The authors did not receive any funding for this study and it was entirely supported by the authors.

## CONFLICT OF INTEREST STATEMENT

The authors declare no competing interests regarding the publication of this article.

## ETHICAL APPROVAL AND CONSENT TO PARTICIPATE

Ethical clearance was obtained from the ethical review board of Jimma University, Institute of Health Science (reference number: IHR 42/2021), and submitted to the Mettu Town Health Office. A letter of permission was obtained from the Mettu Town Health Office. Before beginning data collection, written consent was obtained from the study participants after clear information about the objectives of the study was provided. For women under the age of 18, appropriate information was provided based on their level of comprehension. Then, an assent, a women's affirmative agreement to participate in the research, was obtained in addition to written consent from their parents. This study was conducted in accordance with the principles of the Declaration of Helsinki. Participants were informed that they had the full right to discontinue or refuse to participate in the study and that any information obtained from them would be kept confidential.

## Data Availability

The datasets used and/or analyzed during the current study will be sent as a Supplementary File if this manuscript is accepted for publication in your journal.
